# High-Resolution Mass Spectrometry-Based Approaches for the Detection and Quantification of Peptidase Activity in Plasma

**DOI:** 10.3390/molecules25184071

**Published:** 2020-09-06

**Authors:** Elisa Maffioli, Zhenze Jiang, Simona Nonnis, Armando Negri, Valentina Romeo, Christopher B. Lietz, Vivian Hook, Giuseppe Ristagno, Giuseppe Baselli, Erik B. Kistler, Federico Aletti, Anthony J. O’Donoghue, Gabriella Tedeschi

**Affiliations:** 1 Department of Veterinary Medicine, University of Milano, 20133 Milano, Italy; elisa.maffioli@unimi.it (E.M.); simona.nonnis@unimi.it (S.N.); armando.negri@unimi.it (A.N.); valentina.romeo@unimi.it (V.R.); 2 Centre for Nanostructured Materials and Interfaces (CIMAINA), University of Milano, 20133 Milano, Italy; 3 Skaggs School of Pharmacy and Pharmaceutical Sciences, University of California San Diego, La Jolla, CA 92093, USA; zhj028@ucsd.edu (Z.J.); christopherbrianlietz@gmail.com (C.B.L.); vhook@ucsd.edu (V.H.); 4 Department of Neurosciences, School of Medicine, University of California San Diego, La Jolla, CA 92093, USA; 5 Department of Pathophysiology and Transplantation, University of Milan, 20133 Milan, Italy; giuseppe.ristagno@marionegri.it; 6 Dipartimento di Elettronica, Informazione e Bioingegneria, Politecnico di Milano, 20133 Milan, Italy; baselli@biomed.polimi.it; 7 Department of Anesthesiology & Critical Care, University of California San Diego, La Jolla, CA 92093, USA; ekistler@health.ucsd.edu; 8 Department of Anesthesiology & Critical Care, VA San Diego HealthCare System, San Diego, CA 92161, USA; 9 Department of Bioengineering, University of California San Diego, La Jolla, CA 92093, USA; federico.aletti@polimi.it

**Keywords:** peptidomics, mass spectrometry, plasma, aminopeptidase, carboxypeptidase, endoprotease

## Abstract

Proteomic technologies have identified 234 peptidases in plasma but little quantitative information about the proteolytic activity has been uncovered. In this study, the substrate profile of plasma proteases was evaluated using two nano-LC-ESI-MS/MS methods. Multiplex substrate profiling by mass spectrometry (MSP-MS) quantifies plasma protease activity in vitro using a global and unbiased library of synthetic peptide reporter substrates, and shotgun peptidomics quantifies protein degradation products that have been generated in vivo by proteases. The two approaches gave complementary results since they both highlight key peptidase activities in plasma including amino- and carboxypeptidases with different substrate specificity profiles. These assays provide a significant advantage over traditional approaches, such as fluorogenic peptide reporter substrates, because they can detect active plasma proteases in a global and unbiased manner, in comparison to detecting select proteases using specific reporter substrates. We discovered that plasma proteins are cleaved by endoproteases and these peptide products are subsequently degraded by amino- and carboxypeptidases. The exopeptidases are more active and stable in plasma and therefore were found to be the most active proteases in the in vitro assay. The protocols presented here set the groundwork for studies to evaluate changes in plasma proteolytic activity in shock.

## 1. Introduction

Recent studies on both human patients and animal models indicate that proteolytic events are involved in the mechanism of multi-organ failure in circulatory shock [1,2,3]. These findings have been considered as an indirect confirmation of the so-called “autodigestion hypothesis”, which implicates that enteral digestive enzymes, released in blood following damage of the intestinal mucosal barrier, play a central role in the progression of shock [4]. In order to gain definitive evidence for the involvement of digestive proteolytic activities in shock, two main conditions must be met: first of all, an increase in the number and quantity of circulating peptides in the blood of shock vs. healthy subjects must be experimentally demonstrated; secondly, proteases/peptidases responsible for generating the peptides must be identified. The latter information would also pave the way for potential therapeutic treatments using specific inhibitors.

The first direct evidence of a statistically significant increase in plasma-circulating peptides following shock was reported in a collaborative effort involving our group using a high-resolution mass spectrometry (HRMS)-based peptidomic approach in rats [1]. The use of animals facilitates the development of various disease models associated with shock under controlled conditions. Peptidase inhibitors can then be evaluated as potential drugs in these animal models. The initial studies were conducted in rats, but a model animal closer to humans is preferred. In accordance, swine models for hemorrhagic and septic shocks have been developed [5,6].

The goal of identifying and quantifying all proteases responsible for the endogenous peptides observed in blood can be tackled using several approaches, each presenting advantages and disadvantages. For example, recent HRMS-based proteomic protocols allow for direct identification and relative quantification of hundreds of peptidases present in blood. The Human Plasma Peptide Atlas 2017 database [7] reports MS evidence for 3509 plasma proteins, of which 234 are peptidases as defined in the MEROPS database [8]. Furthermore, according to annotation in the Gene Ontology (GO) database [9] using “peptidase” or “protease” as keywords, 436 out of the 3509 plasma proteins are directly related to proteolysis. Focusing on exopeptidases considered as “canonical” components by the Human Plasma Peptide Atlas 2017 database, according to GO annotation, human plasma contains 34 aminopeptidases and 25 carboxypeptidases.

However, direct identification of peptidases by proteomics does not give quantitative information about the enzymatic activity in plasma. Peptidomics is an indirect method to detect and quantify peptidase activity as peptides derived from precursor proteins can only be generated by the action of one or more peptidases. Therefore, comparing the plasma peptide sequence and abundance in animal models of disease to healthy controls allows for indirect quantification of protease activity that is associated with the disease. In addition, this powerful approach allows for the identification and relative quantification of important biologically active peptides under different conditions.

While peptidomics provide us with a way to detect protease activity that has occurred in plasma, it would be ideal to correlate this activity with a method to directly quantify protease activity in plasma. We have developed an assay called multiplex substrate profiling by mass spectrometry (MSP-MS) to uncover the proteolytic activity of complex biological samples in a global and unbiased manner [10,11]. This assay uses a library of 228 synthetic reporter peptides to assess the substrate specificity and kinetic efficiency of all proteases in parallel, including aminopeptidases, endoproteases and carboxypeptidases. Previously, we used MSP-MS to uncover the peptide cleavage preferences of proteases in neutrophil extracellular traps. These studies determined that neutrophil elastase is the dominant protease activity associated with the neutrophil extracellular traps [12]. In addition, the global proteolytic activity in premalignant pancreatic cyst fluid was revealed by MSP-MS and shown to be significantly different from the proteolytic activity of benign cyst fluid. Fluorogenic substrates were designed to evaluate these differences in a microplate assay format [13]. Using cyst fluid samples from 110 patients, one fluorogenic substrate was rapidly cleaved by the aspartic acid protease, gastricsin, in premalignant cysts and differentiated from benign cysts with 100% specificity and 93% sensitivity. In general, we can calculate catalytic efficiency (k_cat_/K_M_) for hundreds of peptide cleavage products in parallel for biological samples and, therefore, using this method to analyze plasma protease activity complements the plasma peptidomics methods that can quantify the changes in concentration of endogenous peptides.

This report highlights the complementary use of peptidomics and MSP-MS to greatly increase our understanding of the basal protease activity in plasma of a porcine animal model. This study sets the groundwork for using these methods to detect and quantify changes in protease activity that are associated with diseases such as septic shock, hemorrhagic shock and cardiovascular disease in general.

## 2. Results

### 2.1. Global Protease Activity Profiling of Pig Plasma by MSP-MS

**Figure 1:**
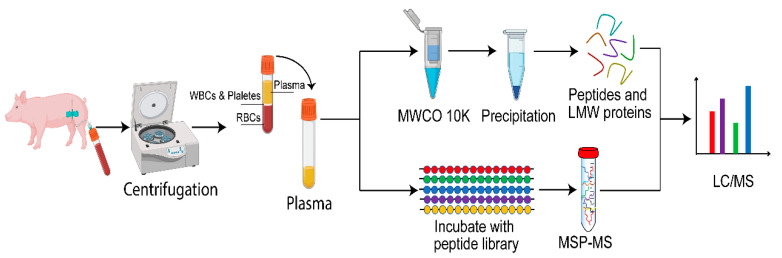
Overview of the protocols applied for the analysis of peptidase activity in pig plasma using multiplex substrate profiling by mass spectrometry assay (MSP-MS) and shotgun label-free quantitative peptidomics.

Plasma peptides from six pigs were separated from plasma proteins by sequential steps of ultrafiltration and precipitation and quantified by liquid chromatography-electrospray ionization-tandem mass spectrometry (LC-ESI-MS/MS). In parallel, proteolytic activity in pooled pig plasma was quantified by incubation with synthetic peptides. The two experimental protocols used in this study are schematically summarized in Figure 1.

To quantify proteolytic activity in plasma samples obtained from healthy pigs, we used the MSP-MS assay, which is a global and unbiased substrate-based protease profiling approach. This assay uses a physicochemically diverse library of peptides as substrates for proteases present in a biological sample. Tandem mass spectrometry is used to quantify protease-derived peptide cleavage products. These substrates were designed to contain a unique dipeptide at each terminus (position 1–2 and 13–14) to profile exo-acting proteases and a central decapeptide sequence (position 3–12) consisting of all combinations of neighbor and near-neighbor amino acid pairs that are used to profile endoproteases [10].

**Figure 2:**
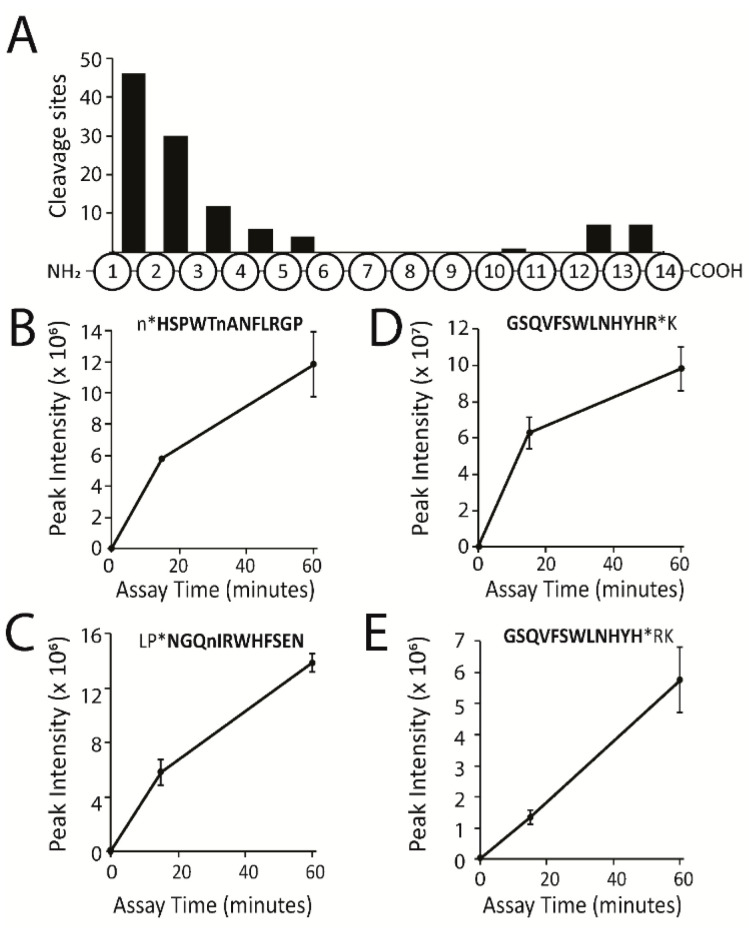
Distribution and quantitation of the cleavage sites within the tetradecapeptide library. (**A**) Distribution of cleavage sites for peptide products that increased by eight-fold or higher after 60 min incubation with *p*-value < 0.05. (**B**) Quantitation of a representative mono-aminopeptidase cleavage product in the control samples (0 min) and following 15 and 60 min incubations. Assays were performed in triplicate. The quantified peptide is highlighted in bold text, lowercase “n” corresponds to norleucine and * is the cleavage site. (**C**) Quantitation of a representative di-aminopeptidase cleavage. (**D**) Quantitation of a representative mono-carboxypeptidase cleavage product. (**E**) Quantitation of a representative di-carboxypeptidase cleavage product.

Prior to incubating the plasma samples with the peptide library, a quality control assay was performed to ensure that each plasma sample contained active proteases. To do this, plasma was diluted 30-fold in assay buffer and incubated with two pools of fluorogenic peptide substrates that have previously been validated as substrates for plasma proteases such as thrombin, plasmin, plasma kallikrein, Factor Xa and urokinase plasminogen activator [14,15,16,17,18,19,20]. These studies confirmed that each of the plasma samples contained active proteases (Figure S1). Equal volumes of plasma from six pigs were mixed and then diluted to a final concentration of 1 mg/mL protein in assay buffer and mixed with the synthetic peptides. This dilution of ~50-fold reduced the concentration of plasma proteins and peptides in the assay that could interfere with the quantification of the synthetic peptides. Cleavage of synthetic peptides by proteases in neat plasma was predicted to occur in minutes and therefore dilution of plasma decreases the concentration of all proteases so that activity could be monitored at two time intervals over the course of an hour-long assay. This allowed us to detect and quantify peptide cleavage products after 15 and 60 min incubations. Under these conditions, 113 cleavage products were quantified, which corresponds to 3.8% of the available peptide bonds (n = 2964) within the peptide library (Table S1). The distribution of these cleavage sites within the 14-residue substrates was evaluated and the highest frequency of cleaved bonds (n = 46) was found to occur at the amino terminus (Figure 2A). Cleavage at this position reveals that mono-aminopeptidases are active in plasma. In Figure 2B, a representative peptide that is cleaved by plasma mono-aminopeptidases is shown, with the peak intensity increasing with time during the assay. The second most frequently cleaved site occurred between the 2nd and 3rd residues at the amino terminal end of the peptide. These bonds are either cleaved by a di-aminopeptidase or are the result of two sequential mono-aminopeptidase cleavage events. Although we cannot rule out that endoproteases may cleave peptides between the 3rd and 6th amino acids, our studies indicate that these truncated peptides are the result of sequential N-terminal trimming by mono- and di-aminopeptidases. For example, the peptide GnYYKRFnAHWVGI is cleaved between Nle and Tyr by a di-aminopeptidase and then further trimmed between Tyr-Tyr, Tyr-Lys and Lys-Arg to yield a 9-mer peptide with the sequence RFnAHWVGI (Table S1).

On the carboxyl terminus, cleavage generally occurs at the peptide bond between amino acids 12–13 and 13–14, which indicates the presence of a mono- and di-carboxypeptidase. A single peptide with the sequence FRIHGFDEAHNAWM was cleaved between His-Asn, which may reveal that an endoprotease hydrolyzes this peptide. However, we also found that this substrate was cleaved between Ala-Trp and therefore it is also possible that the Trp-Asn cleavage is the result of sequential processing by a di-carboxypeptidase (Table S1). Taken together, these data show that under these assay conditions, protease activity in pig plasma is dominated by aminopeptidases and carboxypeptidases.

**Figure 3:**
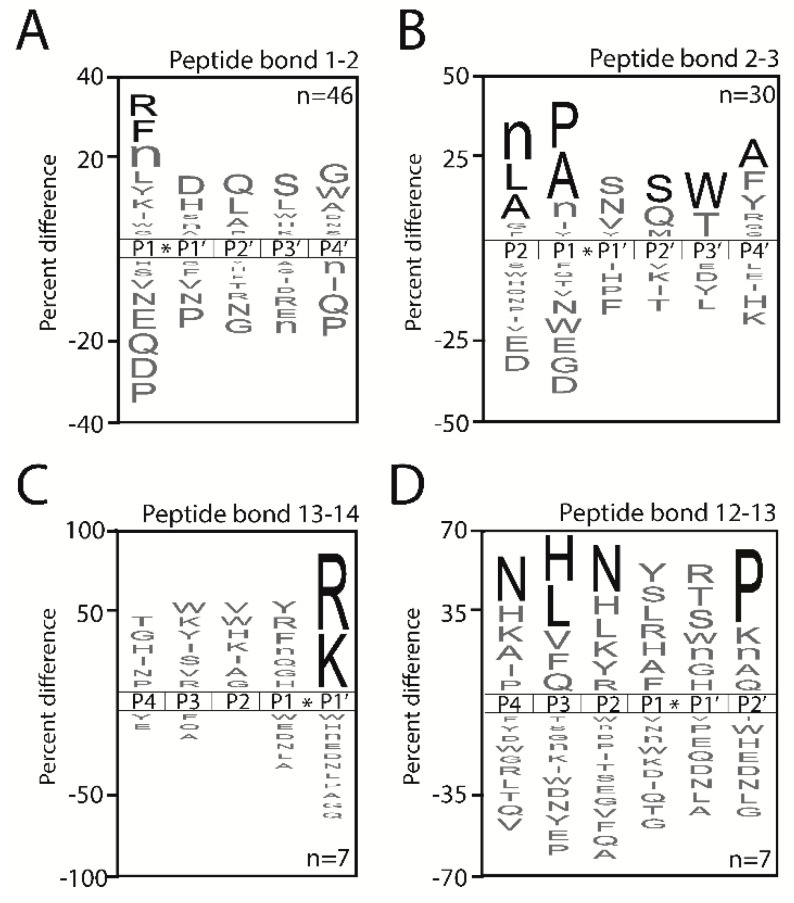
Substrate specificity pattern of pig plasma samples determined using multiplex substrate profiling by mass spectrometry. (**A**–**D**) The iceLogo plots represent amino acids that are most frequently above the axis and least frequently below the axis when observed in the cleavage sites [21]. The time at which the cleavage was observed is indicated at the top of each plot. Residues shown in black are significantly (*p* < 0.05) increased in frequency while residues in gray have *p* > 0.05. Lowercase n corresponds to norleucine while the number of cleavage sites used to make the iceLogo plots are indicated in the bottom right corner of each panel.

To characterize the aminopeptidase and carboxypeptidase activity, we generated iceLogo frequency plots [21] illustrating the substrate specificity pattern of all peptide cleavages detected in pig plasma samples after 60 min of incubation. The MPS-MS study identified five unique protease activities in the plasma samples, all of which displayed exopeptidase activity. After 60 min of incubation, we uncovered 46 cleavage sites generated by plasma proteases that are located at the amino terminus, between residues 1–2. We predict that these sites are generated by two distinct mono-aminopeptidases; one that prefers to cleave after hydrophobic amino acids (Leu, Nle, Phe, Tyr, Ile) and one that catalyzes the cleavage of basic residues (Arg, Lys) (Figure 3A). It is unlikely that a single aminopeptidase can cleave this diverse set of N-terminal amino acids. Peptides that have an N-terminal amino acid such as Pro, Asp, Gln, Glu or Asn are rarely or never cleaved by a mono-aminopeptidase.

At the peptide bond located between residues 2 and 3, we detected 30 unique cleavage sites that are generated by one or more di-aminopeptidases. In general, dipeptides that are removed consist of hydrophobic residues, such as Leu-Pro, Ala-Ala, Ala-Pro and Nle-Ala, at the amino terminus, while peptides that are N-terminally capped with Asp or Glu in the 1st or 2nd position are never cleaved. In addition, peptides with Gly, Trp or Asn in the 2nd position are also never or rarely cleaved. For this di-aminopeptdase activity, there is a significant enrichment of substrates with Ser at P2′, Trp at P3′ and Ala at P4′. (Figure 3B). The MSP-MS profiling also showed that a di-carboxypeptidase in plasma removes dipeptides from the C-terminus (peptide bond 12–13) when Pro is the terminal residue (Figure 3C). Peptides that have other amino acids in this position, such as Lys, Nle, Ala and Gln, are also frequently cleaved, however, none of these amino acids are significantly (*p* < 0.05) enriched at this position. Peptides containing Asn in the P2 and P4 position and His or Leu in the P3 position are also frequently cleaved. Under these conditions, a total of seven unique cleavage sites were identified in the peptide bond 13–14, and the substrate signature revealed the presence of a very strong mono-carboxypeptidase that exclusively removes C-terminal Lys and Arg residues (Figure 3D).

Collectively, our data confirmed that the peptide library is broadly applicable for profiling numerous proteases in pig plasma samples; however, under the conditions of the assay, only exo-peptidase activity was detected.

### 2.2. Peptidomics of Pig Plasma

A shotgun label-free quantitative peptidomic approach was applied to investigate the plasma peptidome of healthy pigs. The workflow included peptide enrichment by ultrafiltration, protein precipitation and centrifugation, LC-ESI-MS/MS analysis, identification, and label-free quantification, as illustrated in Figure 1. Analyses were performed on six biological replicates.

**Figure 4:**
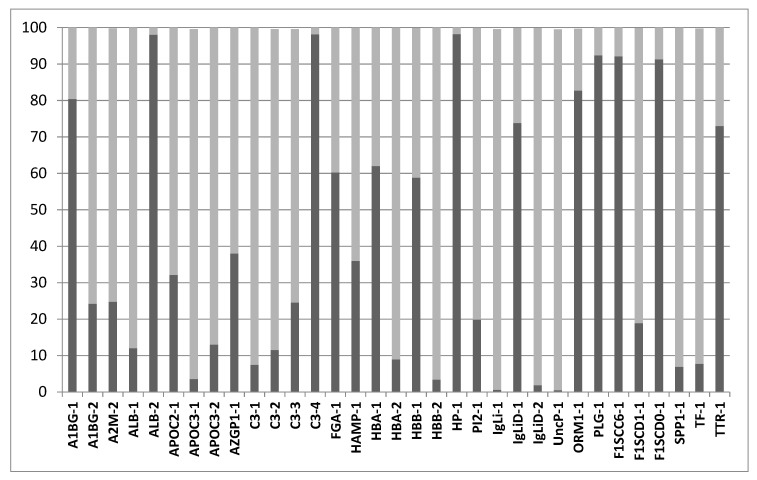
Relative percentage of forms deriving from endopeptidase (light gray) or endopeptidase followed by exopeptidase (dark gray) forms of the peptides undergoing endo- and exopeptidase cleavages listed in Table S2.

Peptidomic analysis allows for the detection and quantification of 172 peptides endogenously produced in swine plasma, originating from the cleavage of 40 proteins (Table S2). Analysis of sequences and positions in the parent proteins reveals that both endo- and exopeptidases are responsible for the generation of the experimentally observed peptides. Table S2 reports experimentally observed 142 endopeptidase and 76 non-redundant exopeptidase cleavages. In particular, when considering the relative abundance, in terms of MS intensity signal, of the endopeptidase followed by exopeptidase generated forms, it can be concluded that a large percentage of extracted current ion intensity in those peptides, which undergo sequential endo-proteolysis followed by exo-proteolysis, is due to peptides generated by endo- and exopeptidase activity. Median values of 25% and 75% are associated with relative percentages of only endopeptidase-derived and endo- and exopeptidase-derived forms, respectively (Figure 4). This is particularly significant since, as discussed below, in the case of endogenous peptides from biological samples, exopeptidase substrates will almost exclusively consist of peptides generated by previous endoproteases, confirming the overall high exopeptidase activity observed in MSP-MS analysis.

**Figure 5:**
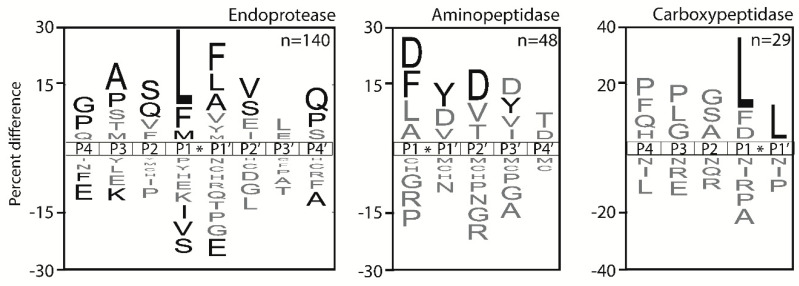
Substrate specificity pattern of pig plasma samples determined using the shotgun mass spectrometric peptidomic approach. The iceLogo plots [21] represent the amino acid frequency surrounding the endo- and exopeptidase cleavage sites using the sequences of non-redundant cleavages (Table S2). Residues shown in black are significantly (*p* < 0.05) increased in frequency while residues in gray have *p* > 0.05. Lowercase n corresponds to norleucine while the number of cleavage sites used to make the iceLogo plots are indicated in the bottom right corner of each panel.

Based on the data listed in Table S2, iceLogo frequency plots [21] were generated to visualize the substrate specificity pattern of non-redundant endo- and exopeptidase cleavages detected in pig plasma samples. (Figure 5).

Concerning endopeptidases, a predominant enrichment of bulky hydrophobic amino acids is observed at positions P1 and P1′, with Leu more favored at P1 and Phe and Tyr at P1′. Conversely, bonds involving polar residues at either position are rarely cleaved. This finding is in agreement with the peptidomics studies in patients and rats performed by our group which showed a prevalence of chymotrypsin-like proteolytic activities in plasma [1,3].

The analysis of the substrate specificity pattern of exo-cleavages has been conducted on the overall amino- or carboxypeptidase activity since, in the case of peptides from animal plasma, it is not possible to distinguish if the forms are generated by a single cleavage of a di- or tri-aminopeptidases or a di-carboxypeptidase or consecutive mono-amino- or mono-carboxypeptidase cleavages. Figure 5 shows two main aminopeptidase activities with preferences for Asp or hydrophobic amino acids at P1 and, probably, a main carboxypeptidase activity with a marked preference for Leu at both positions flanking the cleaved bond.

## 3. Discussion

In this study, our goal was to perform unbiased substrate profiling of proteases in plasma from healthy animals in order to develop and validate protocols for studying changes in plasma protease activity and specificity in patients and animal models with circulatory shock due to trauma and hemorrhage or infection. Several studies have previously quantified protease activity in plasma using reporter substrates that are cleaved by endoproteases with a preference for arginine, phenylalanine, proline and leucine in the P1 position [22,23,24,25]. In addition, endoprotease activity was detected by monitoring the rate of dye released from protein substrates such as casein or gelatin as they are degraded [22,26] For studying plasma proteases systemically active in shock, assays should be sensitive enough to detect subtle changes in activity and the reporter substrates need to be sufficiently diverse to detect aminopeptidases, carboxypeptidases and endoproteases. To do this, we have employed two complementary methods that each utilize nano-LC-ESI MS/MS. Shotgun peptidomics quantifies protein degradation products that have been generated in vivo by proteases, while MSP-MS quantifies plasma protease activity in vitro using a global and unbiased library of synthetic peptide reporter substrates. Although both MSP-MS and shotgun peptidomics are high throughput methods relying on nano-LC-ESI MS/MS, the two approaches are distinct regarding the type of information provided from the same sample. The aim of the current study is to examine the basal protease activity in the plasma of healthy pigs by integrating the information obtained from peptidomics and MSP-MS with the goal of standardizing these methods so that we can readily quantify changes in protease activity associated with disease. For example, we and others have ongoing studies in animal models of hemorrhagic shock, septic shock, fungal infections [27] and preclampsia [28], all of which involve changes in plasma protease activity. This study showed that a diverse range of peptides are generated by endo- and exopeptidases in plasma and the enzymatic activity can be directly quantified in vitro using a library of diverse synthetic peptides.

Comparing the results from the two approaches, it can be concluded that the specific activity of exopeptidases in plasma is higher than the specific activity of endopeptidases. However, these endopeptidases are clearly active in plasma, as they generate peptides from the interior of plasma proteins, which is something that cannot be performed by exopeptidases. When plasma was diluted 50-fold and incubated with the MSP-MS assay for 15 min, we detected cleavage of many peptides in the library but only at sites near the amino and carboxy terminus. After 45 additional minutes of incubation, the relative abundance of cleavage products increased further, revealing that the exopeptidases retain activity for at least one hour under these in vitro assay conditions. The high ratio of exopeptidase activity in complex biological samples relative to endopeptidase activity has previously be shown by our group for human lung cancer cell secretions [11], while other complex samples, such as fungal extracts, have strong endopeptidase and carboxypeptidase activity with no aminopeptidases [11]. Other samples, such as the midguts of beetles, have mostly endopeptidase activity and little exopeptidase activity [29]. When complex biological samples, such as plasma, have a strong exopeptidase component, it decreases our ability to detect endopeptidase activity. This is due to competition between multiple enzymes for the same substrates, resulting in a rapid decrease in substrate concentration with time. To overcome this, our future studies will evaluate endoproteases and exoproteases separately, by including inhibitors of each enzyme group in the assay.

The final goal of the study was not to generate a complete quantitative description of serum peptidome but to develop and validate methods suitable for quantifying differences in plasma proteolytic activities between healthy and shock subjects and also between untreated and drug-treated animals. A major limitation of the shotgun peptidomic approach concerns its sensitivity, due to technical difficulties in the identification/quantification of endogenous peptides present in low amounts. This limitation, which is a common issue in this type of direct unbiased MS approach, could be partially overcome in future experiments by labeling peptides with ionization enhancers prior to MS analysis, as reported in [30].

Combining information on the largest current set of pig plasma proteins from previous proteomics studies [31] with annotations of pig proteins in the Gene Ontology database [9] reveals that there are at least 115 peptidases in pig plasma, many of which have been defined as endo- or exopeptidases based on their sequence homology to related enzymes. In addition, 38 peptidase inhibitors have been identified. While these proteomic studies may provide an approximation of relative abundance of each enzyme and inhibitor, it cannot be used to determine which peptidases are catalytically active and which peptidases have been inactivated by inhibitors. It is possible that the half-life of endopeptidases in plasma is considerably shorter than the half-life of exopeptidases due to rapid inactivation of endopeptidases by inhibitors such as alpha-2 macroglobulin, alpha-1-antitrypsin and alpha-1-antichymotrypsin. Therefore, these enzymes are not detected in the in vitro assay but the peptidomics data reveal that they were previously active in the plasma. In both assays, abundant exopeptidase activity was detected. In the peptidomics study, exopeptidases often generated overlapping “families” of peptides corresponding to the sequential degradation of specific regions of proteins. Prior to cleavage by the endopeptidases, these regions could not be cleaved by exopeptidases because they were distal from the amino and carboxy terminus of the full-length protein substrate. Exopeptidases are crucial components of the blood proteome, playing key roles in the synthesis and metabolism of a number of biologically active peptides involved in many physiological and pathological processes, such as blood pressure, coagulation, control and innate immunity regulation, as well as metabolic diseases and cancer [32]. In this regard, the key role of ACE2 carboxypeptidase as the receptor of the COVID-19 spike protein is well known [33].

Concerning statistically enriched cleavage specificity, the two methods detect a common aminopeptidase activity specific to hydrophobic residues at P1, while the specificity of the main carboxypeptidases is completely different, since peptidomics show a clear preference for hydrophobic residues at P1-P1′, while MSP-MS detects an activity with no clear preferences at P1 but is absolutely specific for basic amino acids at P1′. Again, this indicates that the results from the two approaches are complementary. Peptidomics indicate that the main endo-proteolytic processes are due to chymotrypsin-like activities, in agreement with the results obtained in patients and in a different animal model [1,3]. Such results are of particular importance, since endo-proteolytic events at hydrophobic amino acids have been shown to be by far the main result of endo-proteolytic activity, which is increased following shock in both systems [1,3]. In summary, this study shows that combining a peptide digestion assay with peptidomics allows us to quantify peptidase activity in vitro and activity that has occurred *in vivo*. These data set the groundwork for future studies that will evaluate changes in proteolytic activity that is associated with disease.

## 4. Materials and Methods

### 4.1. Plasma Sample Preparation

Six milliliters of arterial blood were withdrawn from the right femoral artery and collected into an EDTA tube. Nine hundred microliters of 7× protease inhibitor solution (Complete Mini (protease inhibitor cocktail), Roche) were immediately added and the blood gently mixed by inverting the tube. Within 10 min, the blood was then centrifuged at 1300 RCF for 10 min at 10 °C and the plasma collected into 0.6 mL aliquots. These aliquots were centrifuged again at 2500 RCF for other 10 min at 10 °C. Plasma was then collected in 0.5 mL aliquots and immediately stored at −80 °C.

### 4.2. Fluorogenic Reporter Assays

Ten fluorogenic peptide reporter substrates each containing a C-terminal reporter group 7-amino-4-methylcoumarin (AMC) were dissolved in DMSO at a concentration of 500 µM. An equal volume of five reporter substrates, namely Z-Arg-Arg-Leu-Arg-AMC (System Peptide Company, Shanghai, China), Boc-Ala-Gly-Pro-Arg-AMC (Enzymes Systems Products, Livermore, CA, USA), N-Benzoyl-Phe-Val-Arg-AMC (Sigma, Burlington, MA, USA), Boc-Leu-Arg-Arg-AMC (Boston Biochem, MA, USA) and Glutaryl-Gly-Arg-AMC (Bachem, Torrance, CA, USA), were combined to form Substrate Pool A. An equal volume of five additional reporter substrates, namely, Arg-AMC, Boc-Val-Arg-AMC, Z-Arg-Arg-AMC, Gly-Arg-AMC (all from Bachem, Torrance, CA, USA) and Z-Phe-Arg-AMC (R&D Systems, Minneapolis, MN, USA), were combined to form Substrate Pool B. Pool A and B were diluted 20-fold in Assay Buffer 1 (Dulbecco’s phosphate-buffered saline, 1 mM DTT, 0.01% Tween-20) such that the final concentration of each substrate was 5 µM. Fifteen microliters of the substrates were added to multiple wells in a black 384-well plate (Thermo Scientific, Waltham, MA, USA, Part # 262260) that contained 15 µL of plasma that was previously diluted 15-fold in Assay Buffer 1. The final composition of each well consisted of five fluorogenic reporter substrates each at 2.5 µM and pig plasma diluted 30-fold in Assay Buffer 1. Fluorescence was measured at 22 °C using a Synergy HTX Multi-Mode Microplate Reader (BioTek, Winooski, VT, USA) with excitation and emission wavelengths of 360 nm and 460 nm, respectively. Protease activity was reported as the change in relative fluorescent units per second. The mean and standard deviation for the activity readings from the six pig plasma samples were calculated.

### 4.3. Peptide Cleavage Site Identification by Multiplex Substrate Profiling (MSP) Mass Spectrometry

The multiplex substrate profiling by mass spectrometry (MSP-MS) assay was performed as described previously with minor modifications [10]. The tetradecapeptide library consists of 228 rationally designed peptides that are each 14 residues in length. The library contains an equal distribution of 18 out of the 20 natural amino acids (no cysteine or methionine) and also contains the non-natural amino acid, norleucine. All neighbor and near-neighbor pairwise combinations of amino acids are present in the library to maximize the number of potential cleavage sites for endoproteases. The amino terminal dipeptide and carboxy terminal dipeptide are distinct in every one of the 228 peptides, therefore providing a highly diverse substrate library for aminopeptidases and carboxypeptidases. The peptides were mixed at an equal molar concentration and diluted in Assay Buffer 2 (Dulbecco’s phosphate-buffered saline, 1 mM DTT) to a concentration of 0.5 µmol/L of each peptide. Six pig plasma samples were pooled and diluted to 2 mg/mL in Assay Buffer 2 and preincubated for 5 min. Thirty microliters of diluted plasma and peptide pools were then combined and incubated at room temperature. Ten microliter aliquots were removed after 15 and 60 min and protease activity quenched with 8 mol/L guanidinium hydrochloride. A control sample consisted of diluted pig plasma mixed with 8 mol/L guanidinium hydrochloride prior to the addition of peptides. The assay was performed in three technical replicates. Samples were desalted with C18 tips and injected into a Q-Exactive Mass Spectrometer (Thermo Fisher Scientific, Waltham, MA, USA) equipped with an Ultimate 3000 HPLC. Peptides were separated by reverse phase chromatography using a C18 column (1.7 μm bead size, 75 μm × 25 cm, 65 °C) at a flow rate of 300 nL/min with a linear gradient of solvent B (0.1% formic acid in acetonitrile) from 5% to 30% with solvent A (0.1% formic acid in water). Survey scans were recorded over a 150–2000 *m/z* range (70,000 resolutions at 200 *m/z*, AGC target 3 × 106, 100 ms maximum).

Tandem mass spectrometry (MS/MS) was performed in data-dependent acquisition mode with higher energy collisional dissociation (HCD) fragmentation (28 normalized collision energy) on the 12 most intense precursor ions (17,500 resolutions at 200 *m/z*, AGC target 1 × 105, 50 ms maximum, dynamic exclusion 20 s). The data were searched against tetradecapeptide library sequences, and a decoy search was conducted with sequences in reverse order with no protease digestion specified. Data were filtered to 1% peptide and protein level false discovery rates with the target–decoy strategy. Peak integration and data analysis were performed using Peaks software (Bioinformatics Solutions Inc.). Peptides were quantified with label free quantification, and data were normalized by LOWESS and filtered by 0.3 peptide quality. Missing and zero values were imputed with random normally distributed numbers in the range of the average of the smallest 5% of the data ± standard deviation (SD).

### 4.4. Nano-LC-ESI-MS/MS Mass Spectrometry-Based Shotgun Peptidomics

Aliquots of 500 µL of plasma samples from healthy (n = 6) pigs were diluted with equal volumes of 32% (*v*/*v*) acetic acid and ultra-filtered using Amicon Ultra-0.5 mL centrifugal filters (MWCO 10K) for high molecular weight protein depletion [2]. The filtrate was then precipitated with two volumes of cold acetonitrile (ACN) containing 0.1% trifluoroacetic acid (TFA) and centrifuged at 13,200 rpm for 30 min at 4 °C to remove residual proteins. The supernatant containing peptides and low molecular weight proteins was collected, dried, dissolved in 1% (*v*/*v*) formic acid and desalted (Zip-Tip C18, Millipore, Billerica, MA, USA) before mass spectrometric (MS) analysis [33].

Nano-HPLC coupled to MS/MS analysis was performed on a Dionex UltiMate 3000 directly connected to an LTQ Orbitrap Velos mass spectrometer (Thermo Fisher Scientific, Waltham, MA, USA) by a nano-electrospray ion source. Peptide mixtures were enriched on 75 μm ID × 200 mm PicoFrit ProteoPrep C18 columns and separated employing the LC gradient: 1% ACN in 0.1% formic acid for 10 min, 1–4% ACN in 0.1% formic acid for 6 min, 4–30% ACN in 0.1% formic acid for 147 min and 30–50% ACN in 0.1% formic for 3 min at a flow rate of 0.3 μL/min. MS spectra of eluting peptides were collected over an *m/z* range of 350–2000 using a resolution setting of 60,000, operating in the data-dependent mode to automatically alternate between Orbitrap-MS and linear ion trap MS/MS acquisition. CID MS/MS spectra were collected for the 20 most abundant ions in each MS scan using a normalized collision energy of 35%, and an isolation window of 3 Da. Rejection of +1 and unassigned charge states was enabled [34]

Raw files from Thermo Xcalibur software (version 2.0) were analyzed using MaxQuant software (version 1.3.0.5) [35] and searched with the Andromeda search engine against the proteome of *Sus scrofa* from the Uniprot database (release 05.10.2016). The initial maximum allowed mass deviation was set to 15 ppm for monoisotopic precursor ions and 0.5 Da for MS/MS peaks. Enzyme specificity was set as unspecific and N-terminal acetylation, methionine oxidation, and asparagine/glutamine deamidation were set as variable modifications. The required false positive rate was set to 5% at the peptide level and 5% at the protein level, and the minimum required peptide length was set to six amino acids.

Only peptides present and quantified in at least four out of six biological replicates were considered as reliably identified and quantified. The mass spectrometry peptidomics data have been deposited in the ProteomeXchange Consortium via the PRIDE [36] partner repository with the dataset identifier PXD008018.

### 4.5. Data Presentation

Information on amino acid sequence patterns preferentially involved in peptidic bond cleavages observed using either direct shotgun peptidomics or multiplex substrate profiling were visualized by iceLogo software (*p* < 0.05) [21]. For multiplex substrate profiling, only peptide sequences that increased in abundance by ≥8-fold after 60 min incubations with *p* < 0.05 (Student’s *t*-test) were considered. For peptidomics analysis, as indicated above, only peptides present and quantified in at least four out of six biological replicates were considered and the *Sus scrofa* genome was used as background.

## 5. Conclusions

This study uses two global and unbiased nano-LC-ESI MS/MS methods to detect active proteases in plasma. Shotgun peptidomics revealed the presence of endopeptidases that cleaved plasma proteins into peptides and exopeptidases that trimmed these peptides on the amino terminus and carboxy terminus. Multiplex substrate profiling by mass spectrometry detected only exopeptidase activity, indicating that these enzymes were more abundant and stable in plasma. Validation of these methods with plasma from healthy animals provides a baseline for studying plasma protease activity in swine models for hemorrhagic and septic shock.

## Supplementary Materials

The following are available online, Figure S1: Preliminary qualitative control assay on samples to ensure the presence of active proteases (such as thrombin, plasmin, plasma kallikrein, Factor Xa and urokinase plasminogen activator). Activity is reported as the change in relative fluorescent units per second. Plasma was incubated with two different mixtures of fluorogenic peptide reporter substrates each containing a C-terminal reporter group 7-amino-4-methylcoumarin (AMC). Detailed composition of Pool A and Pool B is reported in Materials and Methods. The mean and standard deviation for the activity readings from the six pig plasma samples are reported. Table S1: Tables reporting cleavages detected in MSP-MS experiments; Table S2: Tables reporting peptides detected in swine plasma samples by shotgun HRMS peptidomics.
