# Supplementary figures and images for: High-Resolution Mass Spectrometry-Based Approaches for the Detection and Quantification of Peptidase Activity in Plasma
Source: Molecules. 2020 Sep 6;25(18):4071. doi: 10.3390/molecules25184071 (PMC7571063; doi:10.3390/molecules25184071)

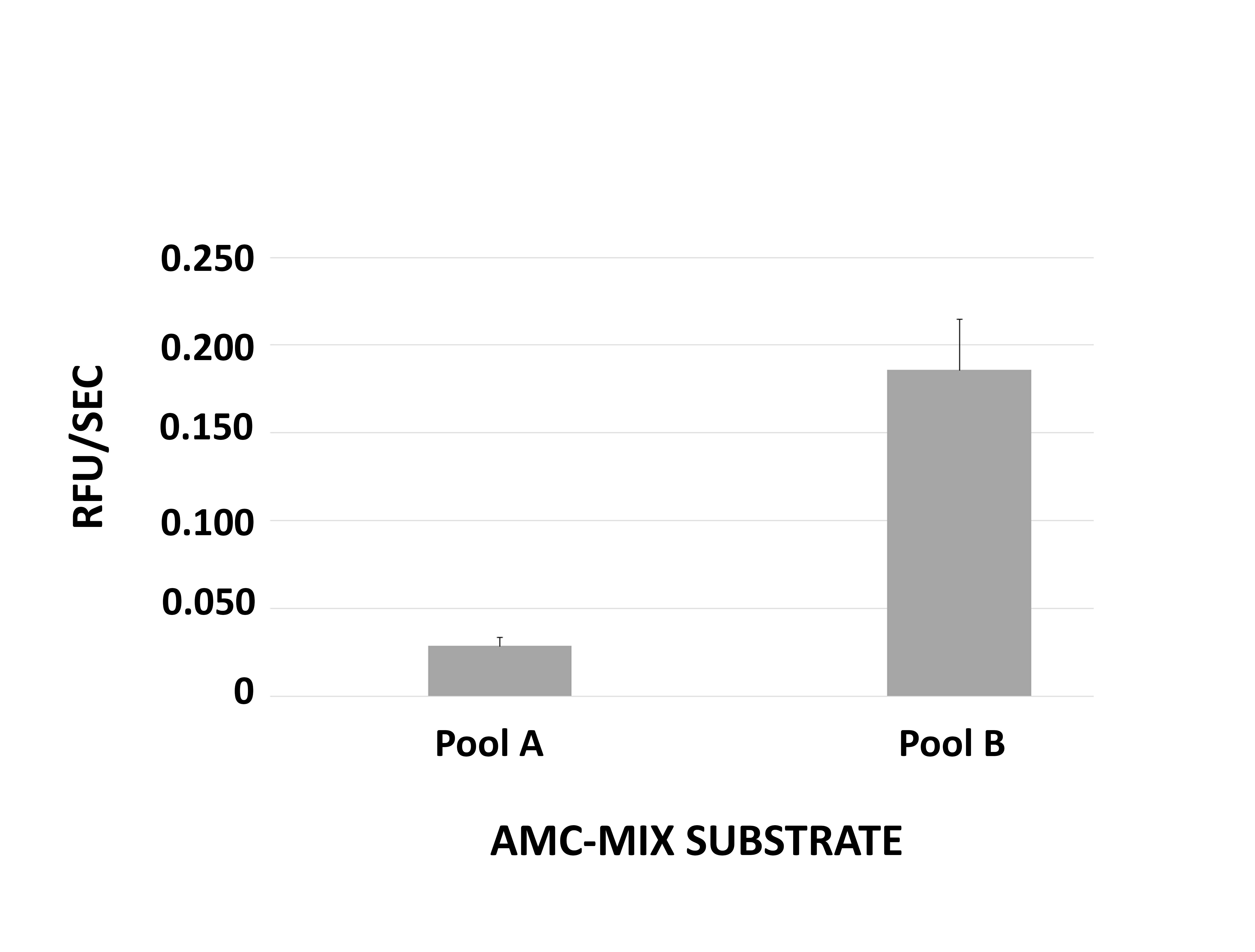

Supplement: Supplementary file 1 [file molecules-25-04071-s001.zip › Figure_S1.jpg]
